# Dimorphic Leaf Development of the Aquatic Plant *Callitriche palustris* L. Through Differential Cell Division and Expansion

**DOI:** 10.3389/fpls.2020.00269

**Published:** 2020-03-10

**Authors:** Hiroyuki Koga, Yuki Doll, Kei Hashimoto, Kiminori Toyooka, Hirokazu Tsukaya

**Affiliations:** ^1^Graduate School of Science, The University of Tokyo, Tokyo, Japan; ^2^RIKEN Center for Sustainable Resource Science, Yokohama, Japan; ^3^Exploratory Research Center on Life and Living Systems, National Institutes of Natural Sciences, Okazaki, Japan

**Keywords:** heterophylly, leaf development, phenotypic plasticity, plant morphogenesis, aquatic plant, *Callitriche palustris*

## Abstract

Heterophylly, or phenotypic plasticity in leaf form, is a remarkable feature of amphibious plants. When the shoots of these plants grow underwater, they often develop surprisingly different leaves from those that emerge in air. Among aquatic plants, it is typical for two or more distinct leaf development processes to be observed in the same individual exposed to different environments. Here, we analyze the developmental processes of heterophylly in the amphibious plant *Callitriche palustris* L. (Plantaginaceae). First, we reliably cultured this species under laboratory conditions and established a laboratory strain. We also established a framework for molecular-based developmental analyses, such as whole-mount *in situ* hybridization. We observed several developmental features of aerial and submerged leaves, including changes in form, stomata and vein formation, and transition of the meristematic zone. Then we defined developmental stages for *C. palustris* leaves. We found that in early stages, aerial and submerged leaf primordia had similar forms, but became discriminable through cell divisions with differential direction, and later became highly distinct via extensive cell elongation in submerged leaf primordia.

## Introduction

Heterophylly is the ability of land plants to produce leaves of different shapes on the same shoot, typically in response to environmental conditions. It is a remarkable feature representing the phenotypic plasticity of plants (Bradshaw, 1965; Zotz et al., 2011). Because plants cannot escape from unfavorable environments, the ability to adapt to such conditions is vital, particularly in habitats characterized by highly variable environmental conditions. Aquatic and riparian environments around ponds, rivers, and wetlands represent highly variable habitats. Water levels may fluctuate seasonally or in response to disturbance events, which leads to flooding and drought risks to plants. Plant species that inhabit these fluctuating environments are referred to as aquatic plants (Cook, 1999). Some such plants are amphibious, which means they can grow in both terrestrial and aquatic habitats. Amphibious plants often exhibit substantial heterophylly as an adaptation to submerged growing conditions (Arber, 1920; Sculthorpe, 1967), and may produce remarkably different leaves depending on if their shoots emerged in air or underwater. Submerged leaves are generally narrower, thinner, and somtimes more branched relative to aerial leaves. These characters are advantageous in submerged conditions (Bradshaw, 1965). Amphibious plants are excellent models for developmental biology studies because the development of two or more leaf forms can be examined in the same individual.

How aquatic plants form such different leaves has been investigated among various eudicots, such as *Ranunculus* species (Ranunculaceae; Young and Horton, 1985; Young et al., 1987; Kim et al., 2018), *Rorippa aquatica* (Brassicaceae; Nakayama et al., 2014), *Hygrophila difformis* (Acanthaceae; Li et al., 2017; Horiguchi et al., 2019), *Ludwigia arcuata* (Onagraceae; Kuwabara et al., 2001, 2003; Kuwabara and Nagata, 2006; Sato et al., 2008), *Rumex palustris* (Polygonaceae; Peeters et al., 2002; Voesenek et al., 2003; Cox et al., 2004, 2006; Vreeburg et al., 2005), *Hippuris vulgaris* (Plantaginaceae; McCully and Dale, 1961; Kane and Albert, 1987; Goliber, 1989; Goliber and Feldman, 1990), and *Callitriche* species (Plantaginaceae; Jones, 1952, 1955a,b; Deschamp and Cooke, 1983, 1984, 1985). In addition, many monocots (e.g., Alismatales and Poales) and even basal angiosperms (i.e., Nymphaeales) are known to show an extensive heterophylly (Arber, 1920; Sculthorpe, 1967), and have been subjected to morphological and physiological studies (Inamdar and Aleykutty, 1979; Titus and Sullivan, 2001; Iida et al., 2009, 2016; He et al., 2018). Because aquatic plants appear in multiple angiosperm lineages, they are thought to have adapted to aquatic habitats independently (Cook, 1999; Du and Wang, 2014). Therefore, heterophylly has also evolved independently, possibly via distinct developmental modifications, and many physiological differences have been reported among aquatic plant species. Various environmental cues, such as light quality and temperature, play different roles in heterophylly among these species (Wells and Pigliucci, 2000). Although the major hormones gibberellin, abscisic acid (ABA), and ethylene are generally involved in the heterophylly among these plants, the roles of these hormones may vary (Wanke, 2011; Nakayama et al., 2017). For example, *R. aquatica* and *H. difformis* show the opposite gibberellin responses (Nakayama et al., 2014; Li et al., 2017). Thus, it is worthwhile to assess adaptations to aquatic environments across various plant lineages. However, only a small number of species have been the focus of modern developmental studies. In this study, we assessed heterophylly in *Callitriche*, a genus that has not been the focus of study in recent decades.

*Callitriche* is a genus of small herbs widely distributed around the world. They are typically aquatic plants, and many have an amphibious form (Erbar and Leins, 2004). The substantial heterophylly exhibited by some *Callitriche* species has been recognized for some time (Schenck, 1886). Thus, *Callitriche* species have been used to examine heterophylly. For example, Jones (1952, 1955a) and Deschamp and Cooke (1983, 1984) utilized *C. intermedia* (a synonym of *C. hamulata*) and *C*. *heterophylla*, respectively, for morphological and physiological studies of heterophylly. They found that osmotic stress can affect submerged leaf formation in both species, and that gibberellin and ABA were involved in heterophylly in *C. heterophylla*. In addition to these works, Jones (1955b) sketched a series of developing leaves of *C. intermedia* and *C. obstagula*. Later, Deschamp and Cooke (1985) described leaf development in *C. heterophylla* mainly by observing cross and longitudinal sections of the shoots. However, possibly due to technical limitations at that time, their collective descriptions were not quantitative or entirely comprehensive.

In this study, we focused on *Callitriche palustris* L., which is closely related to *C*. *heterophylla* (Philbrick and Les, 2000; Ito et al., 2017). Although *C*. *palustris* exhibits extensive heterophylly associated with submergence (Schenck, 1886; Arber, 1920), heterophyllous leaf development has not yet been described in this species. We systematically described and categorized leaf morphogenesis in this species and provide a comprehensive reference for further developmental studies.

## Materials and Methods

### Plant Cultures and Transplanting

*Callitriche palustris* plants were collected in Hakuba, Nagano Prefecture, Japan, and cultured on soil (Aqua Soil Amazonia, Aqua Design Amano, Japan) in a growth chamber with long-day conditions (16 h light and 8 h dark) at 23°C with a light intensity of 60 μmol m^–2^ s^–1^. Plants were also cultured under sterile conditions with half-strength Murashige and Skoog (1/2 MS) salt (Wako Pure Chemical Industries Ltd., Osaka, Japan) containing 2% (w/w) sucrose and 0.3% (w/w) gellan gum (Wako Pure Chemical Industries Ltd).

Shoots were dissected from sterile plants for use in leaf development analyses. To eliminate preexisting leaf primordia, visible shoot tips were removed during transplanting (Supplementary Figure S1A). Then transplants with two or three nodes were transplanted into a plastic container (Incu Tissue PC; SPL Life Science Co., Ltd., Pocheon, South Korea) with 50 mL solidified culture medium and cultivated in the growth chamber. For submerged growth experiments, the container was filled with 200 mL sterile distilled water (Supplementary Figure S1A). After 2 to 3 weeks, axillary shoots that had emerged from transplant nodes were used for further experiments and measurements.

### Leaf Measurements

Prior to analyses, plant shoots were fixed with formalin-acetic acid-alcohol (FAA) fixative. Leaves were dissected from these fixed shoots and images were taken with a scanner or a camera attached to a microscope. Then measurements were obtained from the scanned images using Fiji/ImageJ software (Schindelin et al., 2012). To measure LMA, 20 fresh mature leaves were dissected from shoots. After scanning to measure leaf area, these leaves were dried and weighed. For cellular observations, fixed leaves were cleared in a chloral hydrate solution (Tsuge et al., 1996). Then leaf cells were observed under a DM4500 differential interference contrast (DIC) microscope (Leica Microsystems, Wetzlar, Germany). Stomatal density of aerial leaves was calculated from multiple microscope images from each leaf. Given that the stomatal distribution was too sparse to count using microscope images, we calculated the stomatal density of submerged leaves by dividing the number of all stomata found on a leaf by the leaf area. For leaf sectioning, fixed leaf samples were embedded in Technovit 7100 resin (Kulzer, Hanau, Germany) as described in Tsukaya et al. (1993), and sectioned into 8 μm thick slices using a rotary microtome (HM360; Thermo Fisher Scientific, Waltham, MA, United States). Sections were stained with 0.1% (w/v) toluidine blue in phosphate buffer saline (PBS), washed with water, dried, and then mounted on glass slides with Entellan^®^ New (Merck Millipore, Burlington, MA, United States). Images were obtained using DIC microscopy (DM4500; Leica Microsystems). Statistical analyses and visualizations were performed using R software.

### Transmission Electron Microscopy Observations

Leaves were cut into two or three pieces and pre-fixed with 2% (v/v) glutaraldehyde and 4% (w/v) paraformaldehyde in 50 mM sodium cacodylate buffer (pH 7.4) overnight at 4°C. After washing with 50 mM sodium cacodylate buffer, samples were post-fixed with 1% (w/v) osmium tetroxide (Electron Microscopy Sciences, Hatfield, PA, United States) in 50 mM cacodylate buffer for 2 h at 21°C. After dehydration in a graded methanol series, the samples were replaced with 100% propylene oxide. Then the samples were infiltrated with Epon 812 resin (TAAB Laboratories Equipment Ltd., Aldermaston, United Kingdom) and embedded. Ultrathin sections (80 nm) were obtained using a diamond knife (DiATOME, Hatfield, PA, United States) on an ultramicrotome (EM UC7; Leica Microsystems) then transferred to Formvar-coated copper one-slot grids. Ultrathin sections were stained with a 4% (w/v) uranyl acetate and lead citrate solution. Images were obtained with a TEM (JEM-1400; JEOL Ltd., Tokyo, Japan) at 80 kV. The thicknesses of the outer cell walls and cuticles were measured from TEM images using Fiji/ImageJ software.

### Flow Cytometry

For flow cytometry analyses, four or five mature leaves from both aerial and submerged shoots were collected. Nuclei were extracted by chopping the leaves with a razor and then stained using propidium iodide as described in Kozuka et al. (2005). Stained samples were analyzed using a BD Accuri C6 Flow Cytometer (Becton Dickinson, Franklin Lakes, NJ, United States).

### 5-Ethynyl-2′-Deoxyuridine (EdU) Incorporation and Signal Detection

The visualization of cell proliferation by EdU was performed using a Click-iT EdU Alexa Fluor 488 Imaging Kit (Thermo Fisher Scientific). Shoot tips were dissected from aerial or submerged plants and then floated on or soaked in distilled water containing EdU, respectively, for 3 h under regular growth conditions to allow for the incorporation of EdU. The detection procedure followed that of Nakayama et al. (2015), but supplementing PBS with 0.1% (v/v) Triton-X100 (Nacalai tesque, Kyoto, Japan). Stained leaves were observed under a DM4500 microscope (Leica Microsystems). For quantitative analyses of EdU signal, EdU incorporation was conducted for 6 h, and the images were taken by a confocal microscope FV10i (Olympus, Tokyo, Japan). Optical stacks for leaf EdU signals were integrated into a single image using the z-stack function with the maximum intensity method; then the signal intensity along the length of the leaf was measured (Supplementary Figure S4) using Fiji/ImageJ software.

### Whole-Mount in situ Hybridization

RNA was extracted from shoots of *C*. *palustris* using an RNeasy plant mini kit (Qiagen, Hilden, Germany). cDNA was reverse-transcribed using PrimeScript (TaKaRa Bio, Inc., Tokyo, Japan). The cDNA sequence of *CpCYCB1*.*1*, with a partial coding sequence, was amplified using the primers 5′–TTCTGTGTGCATTCCATCAAAATCTC–3′ and 5′–CTGAAAAACCAGTATGCAGTTTCAATG–3′. The amplified fragment was cloned into pZErO-2 vector. The sequence was deposited in DNA Data Bank of Japan (Accession number: LC516419). The template for probe synthesis was amplified by PCR using M13F and M13R primers, and digoxygenin (DIG)-labeled RNA probes were transcribed using a DIG labeling RNA kit (Roche, Basel, Switzerland). For whole-mount *in situ* hybridization, we followed Rozier et al. (2014) with slight modifications. Briefly, we modified the fixative to 4% (w/v) PFA (TAAB) with 1% (v/v) glutaraldehyde (TAAB) in PBS supplemented with 0.1% (v/v) Tween-20 (Nacalai tesque), and changed the cell wall enzyme treatment to triplicate concentrated solution for 30 min at 42°C. Images were obtained by microscopy (DM4500; Leica Microsystems).

### Phylogenetic Analyses

Protein sequences similar to the proteins of interest were obtained by conducting BLASTp searches against protein databases. Sequences were aligned using the MAFFT program (v.7.429, Katoh and Toh, 2008) and low-homology regions were trimmed using trimAl (v. 1.4, program automated-1; Capella-Gutiérrez et al., 2009). Maximum-likelihood trees were constructed using RAxML (v.8.2.12; Stamatakis, 2014) with 1,000 bootstraps.

## Results and Discussion

### Establishment of a Laboratory Strain of *C. palustris*

*Callitriche palustris* is among the most widely distributed species of the genus *Callitriche*. It occurs across the Northern Hemisphere, including Asia, Europe, and North America (gbif.org). We collected wild *C*. *palustris* from Hakuba, Nagano Prefecture, Japan, which we named “NH1” after the collection location, and established a culture system in the laboratory using this strain. We found that the plants grew well and could be constantly harvested from the growth chamber. We confirmed that germination in *C*. *palustris* can be induced by soaking the seeds in freshwater. Furthermore, plants could be grown on 1/2 MS medium under sterile conditions as well as on soil, and the first batch of fruits were mature only 2 months after germination. We used plants grown under sterile conditions in MS medium to better control the culturing environment, namely, the nutrient supply, and to avoid any effects from microbial activity. Experimental plants were clones of the same individual, wherein stems with nodes were transplanted to either aerial or submerged growing conditions. As the transplanted nodes bore new axillary shoots, the shoots eventually formed aerial or submerged leaves depending on their environment (Supplementary Figure S1 and Supplementary Videos S1, S2).

### Mature Leaf Morphology

**FIGURE 1:**
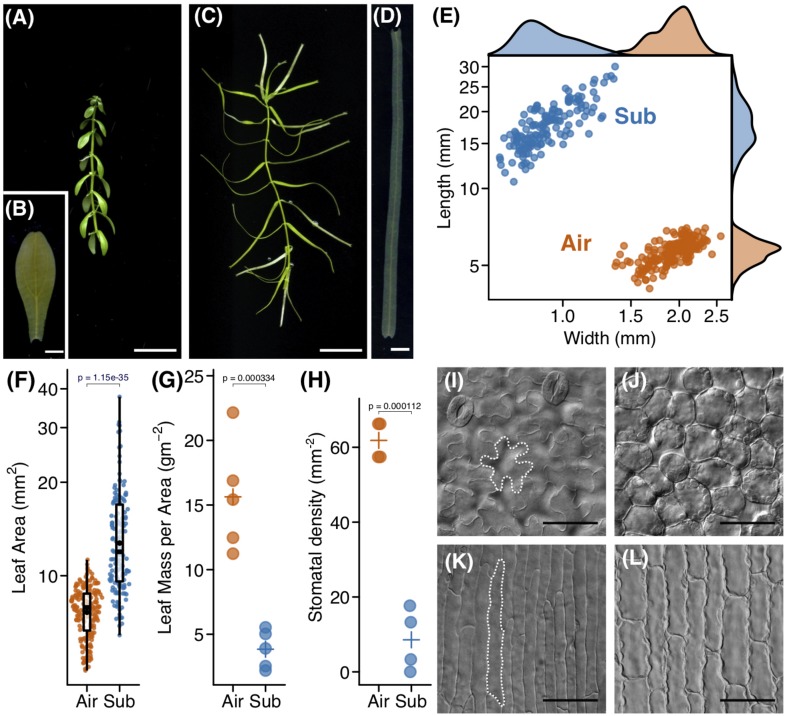
Heterophylly of *Callitriche palustris*. **(A)** Appearance of an aerial shoot and **(B)** its mature leaf. **(C)** Appearance of a submerged shoot and **(D)** its mature leaf. **(E)** Plot showing the length and width of mature leaves. Each point indicates a mature leaf. **(F)** Plot of leaf area. Color points indicate individual leaves and black points indicate mean values. A *P*-value was calculated using Welch’s *t*-test with log–transformed values. Plots of **(G)** leaf mass per area and **(H)** stomatal density. Color points indicate individual leaves and crosses indicate mean values. *P*-values were calculated using Welch’s *t*-test. Paradermal view of cells of **(I,J)** an aerial, and **(K,L)** a submerged leaf. **(I,K)** adaxial epidermal layer and **(J,L)** sub–epidermal palisade layer **(J,L)**. Scale bars: **(A,C)** 1 cm, **(B,D)** 1 mm, **(I–L)** 50 μm.

To investigate leaf development in *C*. *palustris*, we first documented the morphologies of mature aerial and submerged leaves. Mature aerial leaves were ovate, whereas submerged leaves were ribbon-like in shape (Figures 1A–D). Leaf measurements demonstrated that submerged leaves were significantly longer and narrower than aerial leaves (Figure 1E). As a result, the ratio of length to width (leaf index) was also substantially higher in submerged leaves. However, the leaf mass per area (LMA) was lower in submerged leaves than in aerial leaves (Figures 1F,G), suggesting that submerged leaves are thinner than aerial leaves. In addition to differences in whole leaf shape, submerged leaves bore characteristic horn-like protrusions at the apex (Figure 1D). An obvious difference was also seen in the number of leaf veins. Nearly all aerial leaves had three veins (176/184; 96.7%), whereas the majority of submerged leaves had a single vein (130/146; 89.0%). Submerged leaves that had three veins were comparable in leaf index to single-vein submerged leaves, although a slight difference in shape was observed between single- and three-veined submerged leaves (Supplementary Figure S2). This suggests that the development of lateral veins is independent of leaf elongation. At the cellular level, jigsaw puzzle-like epidermal cells and globular palisade cells were observed from the paradermal view in aerial leaves (Figures 1I,K). By contrast, drastic elongation along the proximal-distal axis was observed in both epidermal and mesophyll cells in submerged leaves (Figures 1J,L). There were also substantial differences in stomata (Figure 1H). In aerial leaves, many stomata differentiated on the adaxial side, but stomatal density on the adaxial side was remarkably lower in submerged leaves.

**FIGURE 2:**
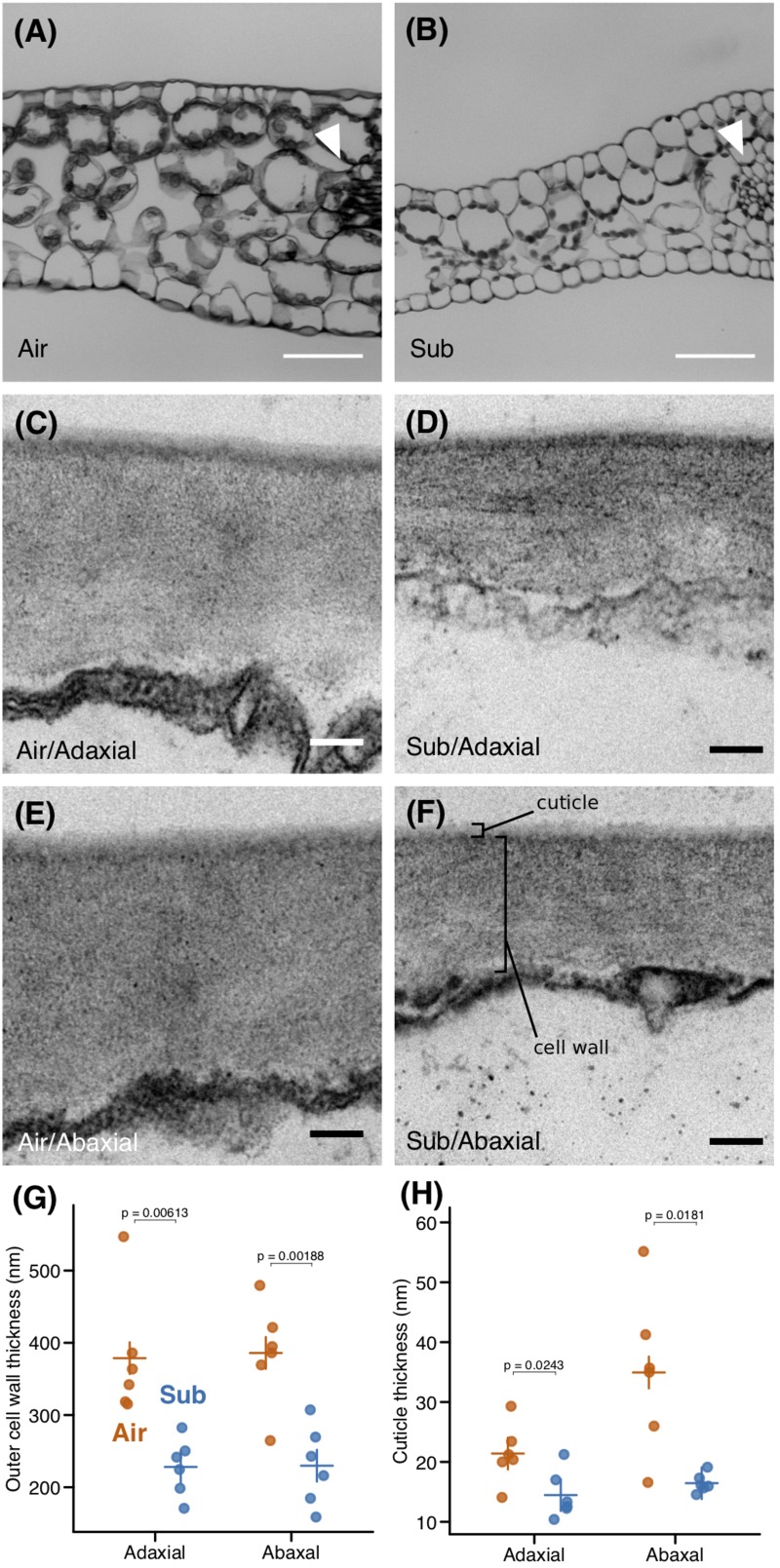
Anatomical differences between aerial and submerged leaves. Transverse sections of **(A)** aerial **(B)** and submerged leaves. Arrowheads point to midveins. Transmission electron microscopy (TEM) images of outer cell walls in **(C,D)** adaxial and **(E,F)** abaxial epidermal cells of **(C,E)** aerial leaves and **(D,F)** submerged leaves. Plots of **(G)** outer cell wall thickness and **(H)** and outer cuticle thickness. Crosses indicate mean values. *P*-values were calculated by Welch’s *t-*test. Scale bars: **(A,B)** 50 μm, **(C–F)** 100 nm.

In examining transverse leaf sections, we did not find any notable differences in leaf layer structure; both leaf types consisted of epidermis, a single palisade layer, and spongy layers along the dorsoventral axis (Figures 2A,B). However, the thicknesses of these layers differed, particularly that of the spongy layer, which may be reflected in the reduced LMA observed in submerged leaves. We measured the thickness of the outer cell wall using transmission electron microscopy (TEM) images and found that the outer cell wall was thinner in submerged leaves (Figures 2C–G). Furthermore, the thickness of the outer cuticle was reduced in submerged leaves (Figure 2H). A reduction in extracellular components may also contribute to the lower LMA of submerged leaves. A reduction of the spongy layer, a thinner outer cell wall, and a reduced or absent cuticle are typical features of submerged leaves of aquatic plants (Sculthorpe, 1967). Thus, the submerged leaves of *C*. *palustris* may follow a common strategy for aquatic plants in underwater environments, adopting modifications that improve processes such as gas exchange in water.

### Aerial and Submerged Leaf Development

**FIGURE 3:**
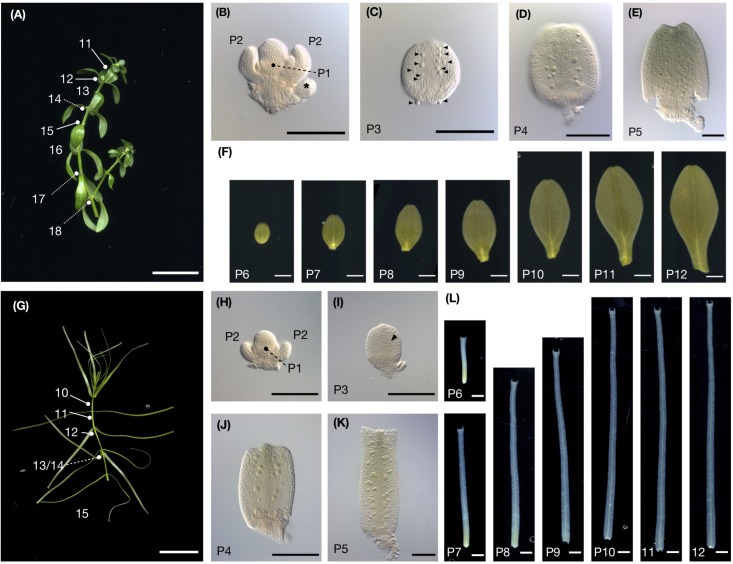
*C*. *palustris* leaf development. **(A)** Shoot grown in air. Node numbers counted from the shoot apex are indicated. **(B)** The shoot apex from panel **A** dissected showing nodes 1 and 2. P1 leaves are borne perpendicular to P2. Asterisks indicate floral buds. **(C–F)** show leave dissected from the shoot shown in panel **A**. **(C)** microscopic image of P3, **(D)** P4, **(E)** P5, and **(F)** scanned images of P6–P12. **(G)** A shoot grown underwater. **(H)** The shoot apex shown in panel G dissected showing nodes 1 and 2. **(I–L)** Leaves dissected from the shoot shown in panel **G**. **(I)** Microscopic image of P3, **(J)** P4, and **(K)** P5, and **(L)** scanned images of P6–P12. Glandular hair cells in **(C,I)** are indicated by arrowheads. Scale bars: **(A,G)** 1 cm, **(B–E, H–K)** 100 μm **(F,L)** 1 mm.

**FIGURE 4:**
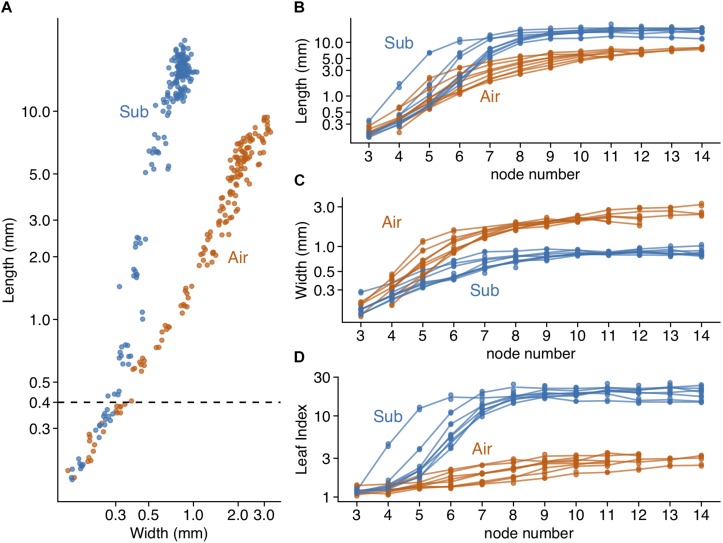
Changes in leaf form during development. **(A)** length-width plot of developing leaves from eight aerial and eight submerged shoots. 164 aerial leaf primordia and 188 submerged leaf primordia are shown as points. **(B)** leaf length, **(C)** Leaf width, and **(D)** leaf index are plotted with node numbers. Lines connect the mean values of pairs of leaf primordia in the same shoot and each point indicates the value of an individual leaf primordium.

We observed leaf morphology across the developing leaf primordia. *C*. *palustris* has decussate phyllotaxy; each node has a pair of leaves. We numbered the nodes from the shoot tip, wherein the smallest pair of leaf primordia adjacent to the shoot apical meristem was node 1, the outer pair was node 2, etc, and referred to the leaf primordia that emerged from these nodes as P1, P2, and so forth, respectively (Figure 3). Then we cut the leaves from the shoots for measurements. In both aerial and submerged shoots, approximately 10 nodes were clustered at the shoot tip, with little internode growth; stem elongations were usually observed below the tenth node (Figure 3). A length-width plot of aerial and submerged leaves indicated distinct developmental trajectories. However, leaf primordia < 400 μm in length did not exhibit clear differences (Figure 4A), consistent with a previous observation in the closely related *C*. *heterophylla* (Deschamp and Cooke, 1985). Differences between aerial and submerged leaf primordia became obvious between P4 and P5. After that stage, primordia on submerged leaves showed biased growth along the proximal-distal axis and an increase in length relative to width, whereas aerial leaf primordia maintained a nearly constant length-width ratio. In both growing conditions, leaves appeared almost mature at P10, at which point we observed a corresponding elongation of the internode stem (Figures 3, 4B–D).

**FIGURE 5:**
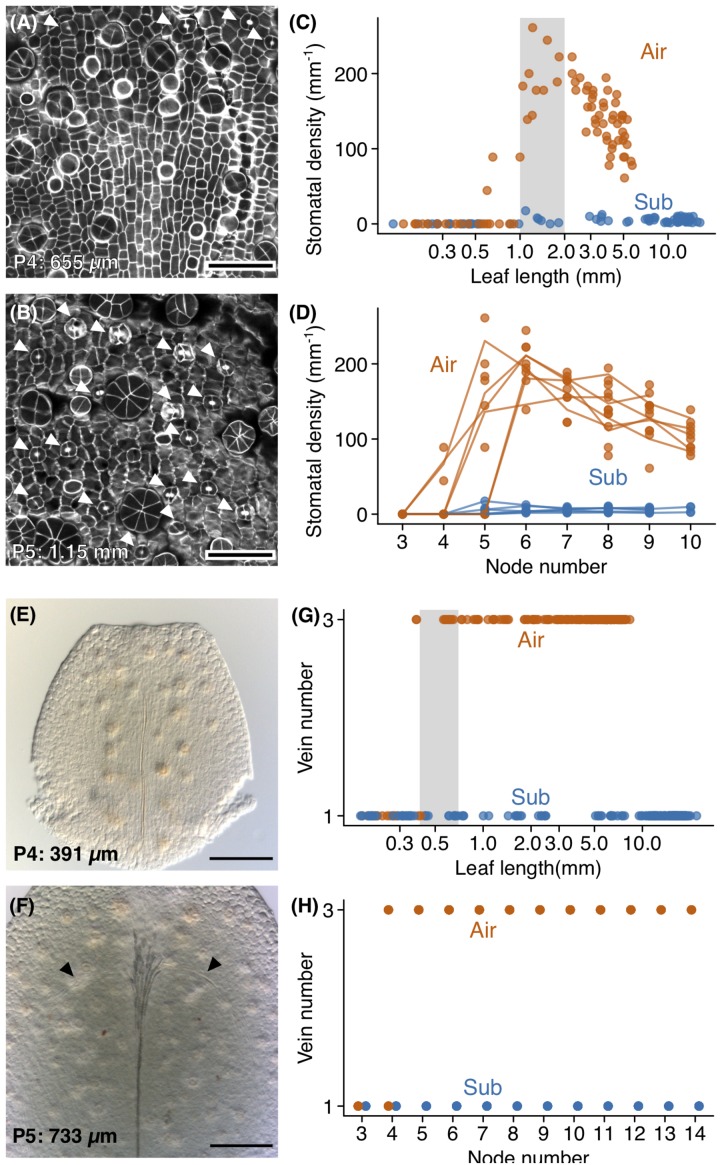
Development of stomata and lateral veins. Confocal images of **(A)** the adaxial epidermis of a P4 aerial leaf primordium and **(B)** the adaxial epidermis from the distal region of a P5 aerial leaf primordium. The top side of the image is the distal side of the leaf primordium. Stomata are indicated by arrowheads, and glandular hair cells appear as circular or radially packed cells. Stomatal density plots of **(C)** leaf length and **(D)** node number. The gray region indicates the range of leaf sizes in which stomata actively differentiated. **(E,F)** Differential interference microscope images of developing aerial leaves. The top side of the image is the distal side of the leaf. **(E)** A P4 aerial leaf with no lateral veins, and **(F)** a P5 aerial leaf showing procambial cells for lateral veins. Procambial cells are indicated by arrowheads. **(G,H)** Plots of vein number against **(C)** leaf length and **(D)** node number. The gray region indicates the range of leaf sizes in which lateral vein differentiations occurred. Scale bars: **(A,B)** 50 μm, **(E,F)** 100 μm. Leaf lengths are shown at the lower left **(A,B,E,F)**.

Stomatal number and lateral venation were significant features differentiating aerial and submerged leaves. Thus, determining when these differences arise is essential to understanding their respective development. Therefore, we observed the timing of stomatal and vasculature development (Figure 5), as well as that of hair cells, which were another tissue present in both leaf types. As noted, stomatal development was extensive on the adaxial surface of aerial leaves but rarely observed in submerged leaves. When observing developing leaves, we found that aerial leaves had active stomatal differentiation during a specific period of development; stomatal density increased when primordia were between 1 and 2 mm in length (P5–P7) and then decreased (Figures 5A–D). The decrease in stomatal density during later developmental stages was likely the result of the expansion of pavement cells, as well as the termination of stomatal differentiation. Furthermore, the stomatal index did not dramatically reduce in the later development period, which supports this explanation (Supplementary Figure S3). Although stomatal density was low in submerged leaves, a small number of stomata had differentiated in primordia >1 mm (P5) and in stages following P5 (Figures 5A–D). The steep increase of stomata in a short developmental period is a notable feature when considering that stomata keep increasing throughout leaf development in Arabidopsis (Asl et al., 2011; Sizani et al., 2018; Challa et al., 2019). Although further investigations and comparisons with relative species are required, it is possible that this feature of stomata development is associated with its amphibious life-type and the drastic plasticity of stomatal development.

Lateral vein development occurred earlier in aerial leaves than did stomatal development and occurred prior to the apparent discrimination of leaf forms. In both submerged and aerial leaves, the midvein developed first (Figure 5E). In aerial leaves, lateral veins developed following midvein development (Figure 5F). Procambial cells for lateral veins were observed in aerial leaf primordia approximately 400 μm in length (Figure 5G). In P4, some leaves had procambial cells for lateral veins, and in P5 all leaves had procambia for three veins (Figures 5F,H). In P6, all leaves had three differentiated veins with lignified xylem (Figure 5H). Therefore, lateral vein differentiation begins in a relatively early phase of leaf development, during which apparent differences in leaf form are not yet established. This observation was consistent with three-veined submerged leaves (Supplementary Figure S2), in that these leaves may have developed their lateral veins prior to the initiation of differentiation in leaf shape. We also note that hair cell differentiation occurred much earlier than the above processes. In both aerial and submerged leaves, hair cells were observed in P3 and P4 leaves (Figures 3C,D).

### The Leaf Meristematic Zone in *C. palustris*

There are two main cellular-level phases of leaf development; cell proliferation and cell expansion. In Arabidopsis and other angiosperms, cell proliferation occurs throughout the entire leaf blade during early developmental phases. Later, the area of active cell proliferation is restricted to the basal region of the leaf blade (Ichihashi et al., 2011; Tsukaya, 2013), wherein cells in the distal region eventually enter the cell expansion phase and those at the base retain meristematic activity for some period of time. The transition from division to expansion is referred to as the cell cycle arrest front, and it recedes toward the base of the leaf blade as development proceeds (Tsukaya, 2005, 2018).

**FIGURE 6:**
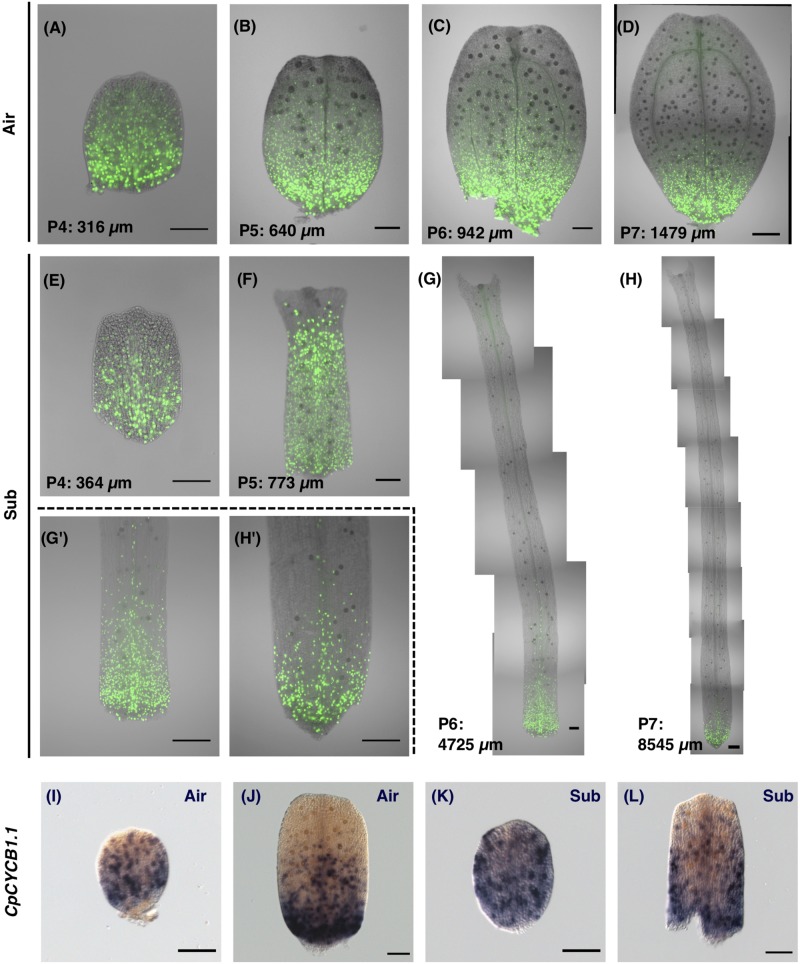
Visualization of the cell proliferation zone by 5-ethynyl-2′-deoxyuridine (EdU) incorporation and *CpCYCB1*.*1* expression. **(A–H)** The cell proliferation zone was visualized by detecting incorporated EdU. **(A–D)** EdU signals in developing aerial leaves. **(E–H)** EdU signals in developing submerged leaves. **(G’,H’)** Magnified images of the basal regions of leaves. **(I–L)** Whole-mount *in situ* hybridization images for the *CpCYCB1*.*1* gene in **(I,F)** aerial leaf primordia, and **(K,L)** submerged leaf primordia. Scale bars: **(A–C,E–G,I–L)** 100 μm and **(D,G,’H,H’)** 200 μm.

Given that differential leaf development in *C*. *palustris* appeared to be associated with different cell expansion processes, it was important to focus upon the developmental period wherein leaf cells enter the expansion phase, to allow for a comparison of leaf development. Thus, we performed 5-ethynyl-2’-deoxyuridine (EdU) incorporation experiments and observed EdU signals in variously sized leaf primordia to determine which areas of leaves were undergoing active cell proliferation (Figures 6A–H). Prior to EdU analyses, we conducted flow cytometry analyses of mature leaves to determine if cells underwent endoreduplication, because endoreduplication in leaf cells can be an additional source of EdU signals to mitotic cell divisions. We confirmed that mature aerial and submerged leaves showed a single main peak of nuclei size (Supplementary Figure S4). Therefore, in *C*. *palustris* leaves, S phase (i.e., the DNA synthesis phase) detection by EdU can be interpreted as evidence of cell proliferation activity.

EdU signals were detected in all regions in early leaf primordia, and during later stages signals were restricted to the basal area of the leaf (Figures 6A–H). Some specific EdU signals in hair cells and vascular cells were observed in more distant regions than the signals in mesophylls and epidermal cells, but these divisions are not likely to have made a direct contribution to leaf expansion. Signals in hair and vascular cells were also restricted to the basal region in late-stage leaf primordia, suggesting that leaf tissues as a whole display acropetal growth.

In addition to the EdU analyses, we examined cell proliferation activity in leaves by investigating the expression of the gene *CpCYCB1*.*1*, an ortholog of *Arabidopsis CYCLIN B1;1/1;2/1;3/1;5* (Supplementary Figure S5). In Arabidopsis, *CYCB1* genes are expressed in cells during the G_2_/M phase and thus are used as a marker of meristematic regions (Donnelly et al., 1999; Kazama et al., 2010; Ichihashi et al., 2011). To analyze gene expression patterns at the whole-leaf scale, we developed methods of whole-mount *in situ* hybridization by optimizing the fixative and treatment conditions of pre-hybridization steps (see section “Materials and Methods”). Then we examined the expression pattern of *CpCYCB1*.*1* in developing leaves and found that gene expression occurs in a salt-and-pepper pattern (Figures 6I–L), which is similar to *CYCB1;1* expression in Arabidopsis, suggesting that this expression marks cells under the mitotic cycle. The expression of *CpCYCB1*.*1* showed a similar pattern to our findings of EdU signals in similarly sized leaf primordia. Thus, we found support that our EdU incorporation and detection method was successful in detecting the meristematic region in leaf primordia.

**FIGURE 7:**
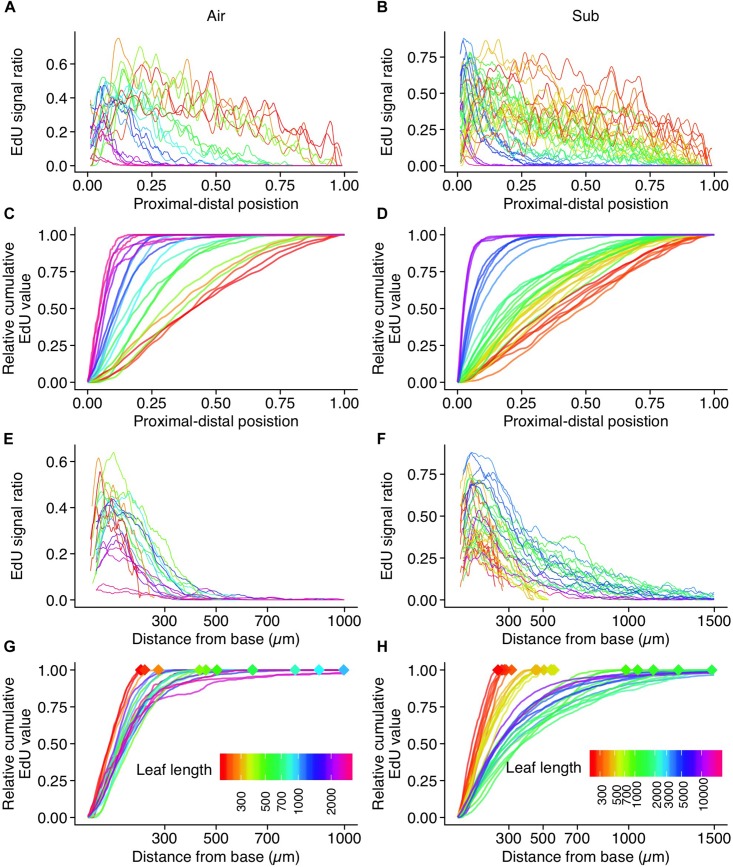
Quantification of EdU signal intensity along the length of a leaf. Plots for **(A,C,E,G)** aerial leaves and **(B,D,F,H)** submerged leaves. Data from 21 and 36 leaves were plotted for aerial and submerged, respectively. **(A,B)** EdU signal ratios are plotted along their relative position to the leaf length. Values were smoothed using the moving average method. **(C,D)** Relative cumulative transformation of plots **(A,B)**, respectively. **(E,F)** EdU signal ratios plotted along the actual distance from the leaf base. Values were smoothed using the moving average method. **(G,H)** Relative cumulative transformation of plots **(E,F)**, respectively. Diamond–shaped points indicate leaf length, which is also shown in different colors. Note that the color codes of leaf length are not shared between aerial and submerged leaf data points.

To quantify cell proliferation throughout leaf development, EdU signal intensity was assessed along the longitudinal leaf axis (Figure 7 and Supplementary Figure S6). We found clear evidence of a negative correlation between leaf length and the relative position of the putative arrest front (Figures 7C,D). In aerial leaves, EdU signals were observed in all regions until leaf primordia reached approximately 300 μm in length. The signals weakened in the distal half of leaf primordia 500–600 μm in length. In aerial leaf primordia approximately 1 mm in length, signals were observed only in the basal-most area, and were further restricted in later development stages. In leaf primordia > 2 mm in length, signal frequency was drastically reduced, indicating that cell proliferation was no longer occurring in leaf primordia of this size (Figures 7A,E). The meristematic zone was ≤500 μm from the primordia base in all developmental stages.

In submerged leaves, EdU signals were also observed in all regions until the leaf primordia reached approximately 300 μm in length. Signals eventually disappeared from the leaf apex once primordia reached 500–700 μm in length. During this stage, the differences in leaf shape between aerial and submerged leaves had become apparent, but the majority of the cells were still in the proliferation phase. This suggests not only that substantial elongation of cells occurs during the expansion phase, but also that a biased direction of cell division contributes to the observed differences in leaf shape between the two leaf types. The shape of submerged leaves suggests that cell division parallel to the proximal-distal axis exceeds that of perpendicular division. Leaf primordia 1–2 mm in length still showed broad cell proliferation. Although a greater signal frequency was observed in the proximal 500 μm region, relatively sparse signals were observed in the distal region (Figure 7F). Close observation indicated that these sparse signals were, in some cases, detected in hair and vascular cells, which was also true of aerial leaf primordia. Given that submerged leaves are narrower than aerial leaves, we would expect a higher fraction of these type of signals along the leaf width. Therefore, background signals in the distal region were conspicuous in the analyses of submerged leaf primordia relative to aerial leaf primordia. Although some signals were observed in mesophyll and pavement cells in the distal region, the main meristematic zone was identified in the basal region within this leaf size (1–2 mm). Frequent signals in the basal region were observed in leaf primordia 5 mm in length, but signal frequency later diminished in primordia > 10 mm. The horn-like structure at the apex of submerged leaves developed in a region with no EdU signal, suggesting that this structure forms through cell expansion. Interestingly, a similar leaf-tip structure was observed in the submerged leaf of *Rotala hippuris* (Lythraceae; Momokawa et al., 2011). This plant belongs to a distant family from *Callitriche*, and thus they have adapted to aquatic environments independently. Although the significance of the leaf-tip structure has never been addressed, the parallel evolution led us to speculate that it has an adaptive function or a developmental consequence of morphological change from ovate aerial leaf to ribbon-shaped submerged leaf.

Leaf meristem position is generally an important determinant of variation in leaf shape (Tsukaya, 2018), but we did not observe major differences in the transitional pattern of the meristematic zone during aerial and submerged leaf development. In both leaf types, meristematic activity was retained in the basal region, approximately 500 μm from the base. As a minor difference, we found that the cell proliferation area was slightly expanded toward the distal end in submerged leaves, which is possibly associated with the exclusive cell division in the proximal-distal direction. Interestingly, after attaining a length of 400–700 μm, the stage wherein differentiation in leaf shape becomes evident, cell proliferation was still active across the leaf primordia. This suggests that changes in cell division patterns play a crucial role in the initiation of differential leaf development. In *Ludwigia arcuata*, it was reported that both differential cell arrangement and cell elongation were involved in the production of the narrower, longer, simple leaves produced in submerged conditions (Sato et al., 2008). Thus, it is possible that there are similar cellular mechanisms at play in submerged leaf formation in *C. palustris* and *L. arcuata*, although these species belong to distant lineages.

### Developmental Stages of *C. palustris* Leaves

**FIGURE 8:**
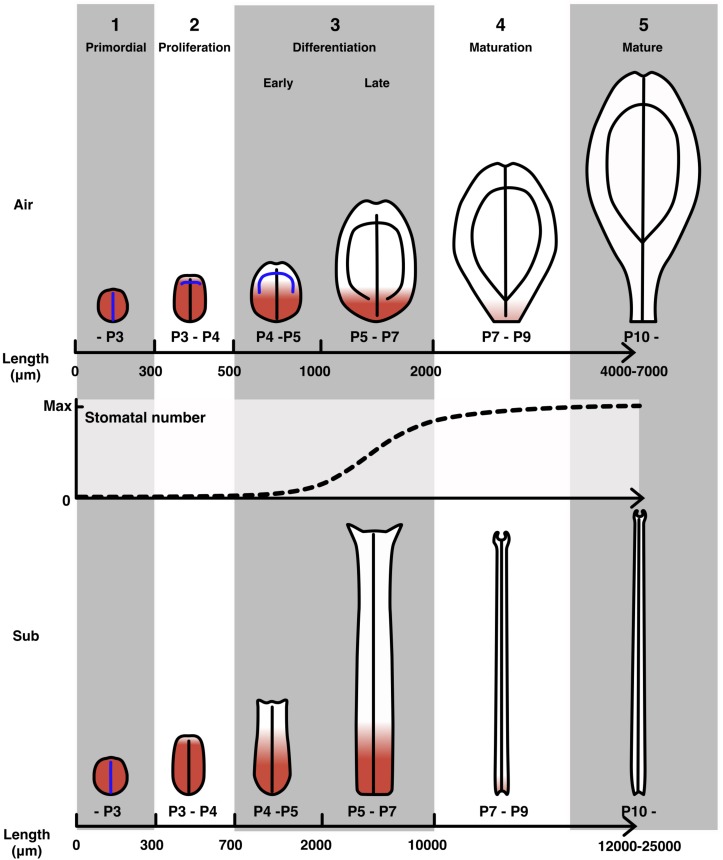
Developmental stages of *C*. *palustris* leaves. Schematic representations of aerial and submerged leaves in each development stage. Red indicates cell proliferation activity. Blue lines indicate undifferentiated vascular tissues, which will become leaf veins as indicated by black lines. Stomatal development in an aerial leaf primordium is displayed as a plot with relative values to the maximum number of stomata in a mature leaf. Note that the scales of the leaf schematics are not uniform and the scales for stages 4 and 5 differ from those of earlier stages.

Using the observations of leaf development described above, we defined developmental stages for *C*. *palustris* leaves (Figure 8). In the first stage, there was little difference in form between aerial and submerged leaf primordia and cell proliferation occurred in all regions of the leaf primordia (Figure 7). No differentiated vascular structures or stomata were observed (Figure 5), but hair cells began to differentiate during this stage (Figure 3). In the second stage, the difference in shape between aerial and submerged leaves, which may be due to differential division, became obvious. Cell proliferation activity was retained in most regions of the leaf primordium, but cells at the distal-most region began to exit the proliferation phase (Figures 6, 7). Procambial differentiation for lateral veins was observed during this stage in some aerial leaves (Figure 5G) but no stomata were differentiated. The third stage is defined as the cell differentiation stage. The relative area of active cell proliferation was reduced in the distal area and eventually retained only in the basal-most part of the leaf primordia (Figures 6, 7). This indicated that a large fraction of the cells in the distal region had begun differentiation and expansion. Differences between aerial and submerged leaves became more distinct throughout this stage. In the early phase of this stage, xylem differentiation of lateral veins proceeded, and in the later phase, stomata differentiation was active in aerial leaves (Figure 5C,G). The fourth stage is a maturation stage wherein no or very few cells proliferated, even in the basal leaf area (Figure 7), but leaves continued to grow via cell expansion (Figure 4). Because distally located cells had entered the expansion phase earlier, those cells may have finished their growth by this stage. Therefore, leaf growth during this stage may be the result of cell expansion in the proximal region. By the end of this stage, cell expansion had ceased and leaves were mature.

We defined developmental stages using leaf length as a developmental index that combined information on tissue differentiation and cell proliferation. However, we note that the leaf lengths provided as stage indicators can vary slightly among shoots, particularly in the late differentiation stage for submerged leaves. Because the length of submerged leaves at maturity varied even in our stable experimental environment (Figure 1E), we suggest that the timing of the exit from cell proliferation and/or the termination of cell expansion may vary among leaves depending on their final size. Nevertheless, in earlier developmental stages, the leaf lengths and developmental processes listed above were well correlated among all observed shoots. Our developmental stage definitions will contribute to further analyses of leaf development, such as gene expression analyses focusing on the transitory time when leaf shape differences become obvious (ca. 400 μm in length), or on differences between the cell proliferation and expansion phases by comparing the apical and basal regions during the differentiation stage.

## Conclusion

We carefully documented and analyzed the process of leaf development in *C*. *palustris* under two different growth conditions, wherein plants bore leaves with highly distinct shapes, and categorized development into five stages. Our observations suggest that during heterophyllous development in *C*. *palustris* differences in both the differentiation processes of each cell (e.g., cell expansion) and cellular arrangement contribute to differential leaf formation. Differences in leaf form were facilitated in the early stages by differential cell arrangements, and by differential cell elongation in the later stages. We found that *C*. *palustris* was a suitable model for modern developmental biology studies, as it was easy to grow in a scalable laboratory environment and has a relatively quick life cycle. This species is also well suited for molecular-based developmental analyses, such as the whole-mount *in situ* hybridization method that we established here. The developmental stages and associated descriptions, as well as the technical foundations of plant culture and developmental analyses, provided by this study will contribute to further understanding of the developmental mechanisms of heterophylly from both molecular and genetic perspectives.

## Data Availability Statement

The raw data supporting the conclusions of this article will be made available by the authors, without undue reservation, to any qualified researcher.

## Author Contributions

HK and HT were responsible for the study design. HK, YD, KH, and KT performed the experiments. HK analyzed the data. All authors contributed to writing and reviewing the manuscript.

## Conflict of Interest

The authors declare that the research was conducted in the absence of any commercial or financial relationships that could be construed as a potential conflict of interest.
