# Supplementary material for: Dimorphic Leaf Development of the Aquatic Plant Callitriche palustris L. Through Differential Cell Division and Expansion
Source: Front Plant Sci. 2020 Mar 10;11:269. doi: 10.3389/fpls.2020.00269 (PMC7076196; doi:10.3389/fpls.2020.00269)
Supplement: Supplementary file 1 [file Image_1.PDF]

Supplementary Material

Supplementary Figures

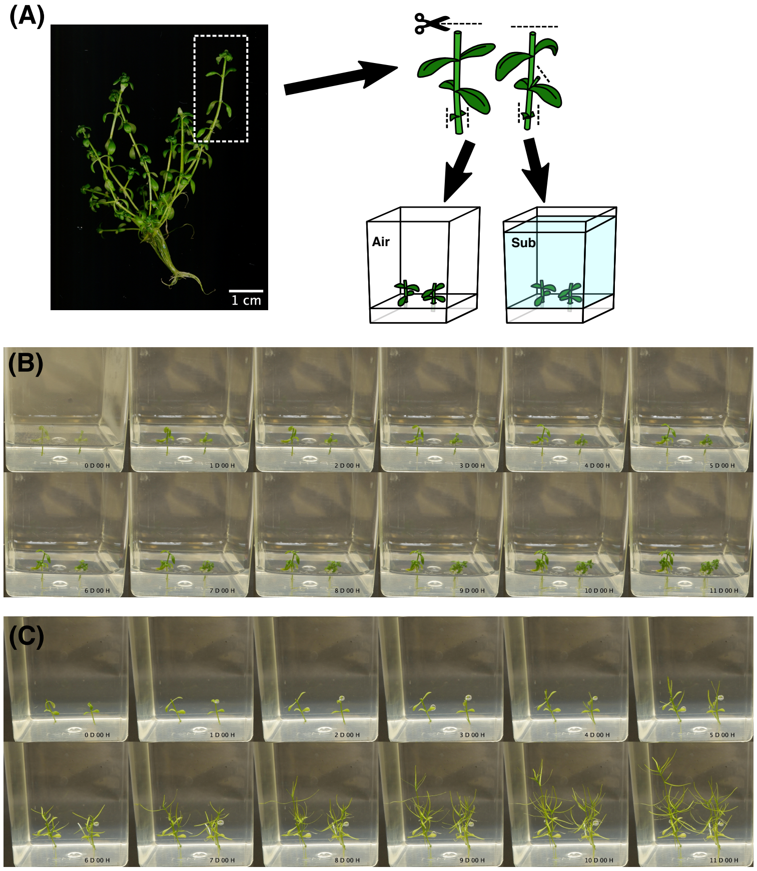

Supplementary Figure 1. Experimental schematic describing growth in *C. palustris*. A) Schematic depicting the growth of *C. palustris* under controlled conditions. Clonal explants were transplanted into different growth conditions. B–C) Time-lapse images of the growth within the aerial B) and submerged C) treatments. Images are shown at one day intervals. Original videos captured at 1 h intervals are available as supplemental videos 1 and 2. Note that aerial plants were exposed to drier than normal conditions during videography to prevent dewing.

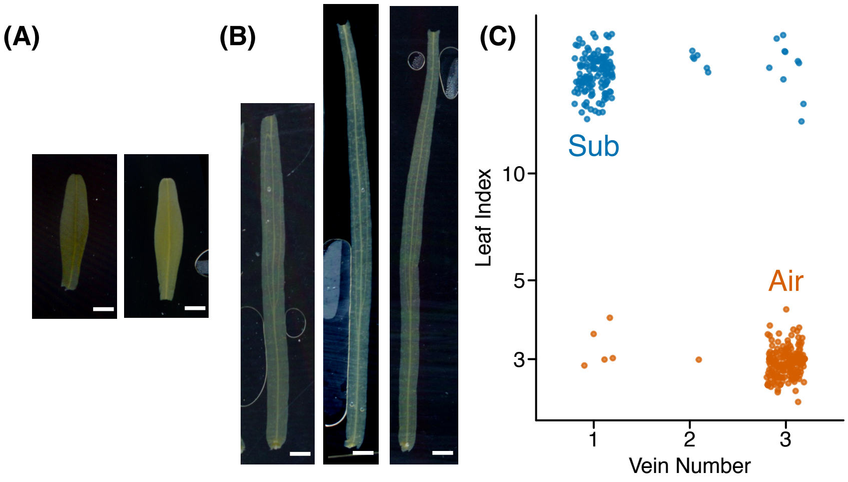

Supplementary Figure 2. **Relationships between vein number and leaf form.** A) Aerial leaves with a single vein. B) Submerged leaves with three veins. C) Plot of vein number and leaf index. Substantial leaf elongation was observed in submerged leaves regardless of the vein number.

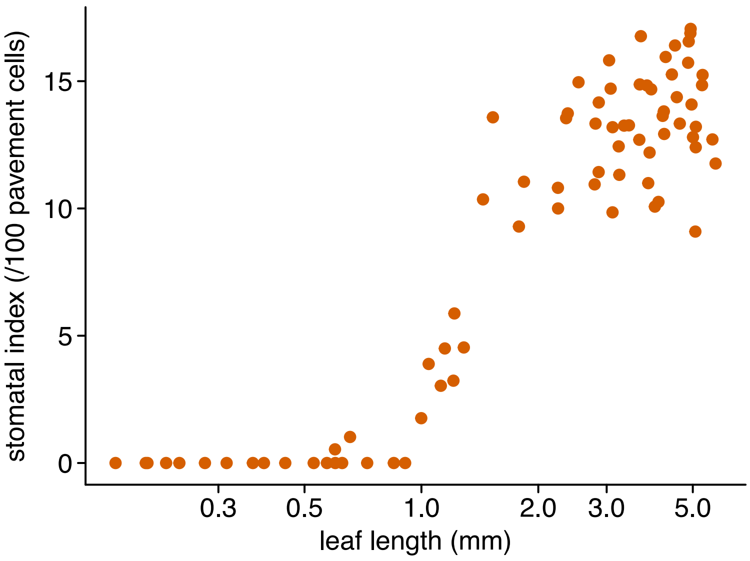

Supplementary Figure 3. Changes in stomatal index during aerial leaf development. Stomatal indices of developing leaves plotted against leaf length.

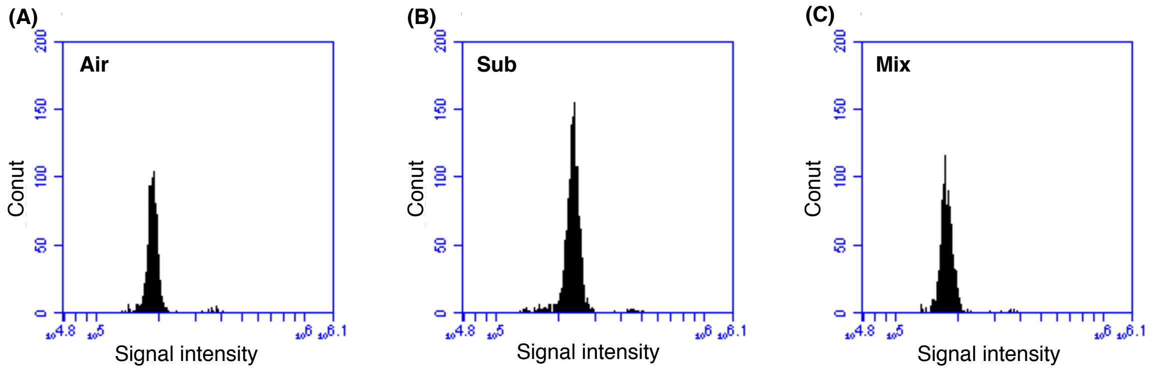

Supplementary Figure 4. Flow cytometry analyses of nuclei from mature leaves. Flow cytometry results of PI-stained nuclei extracted from A) aerial leaves, B) submerged leaves, and C) a mixed sample of aerial and submerged leaves. In all cases, an identical single peak was detected, indicating a uniform ploidy level among leaf cells and between growth conditions.

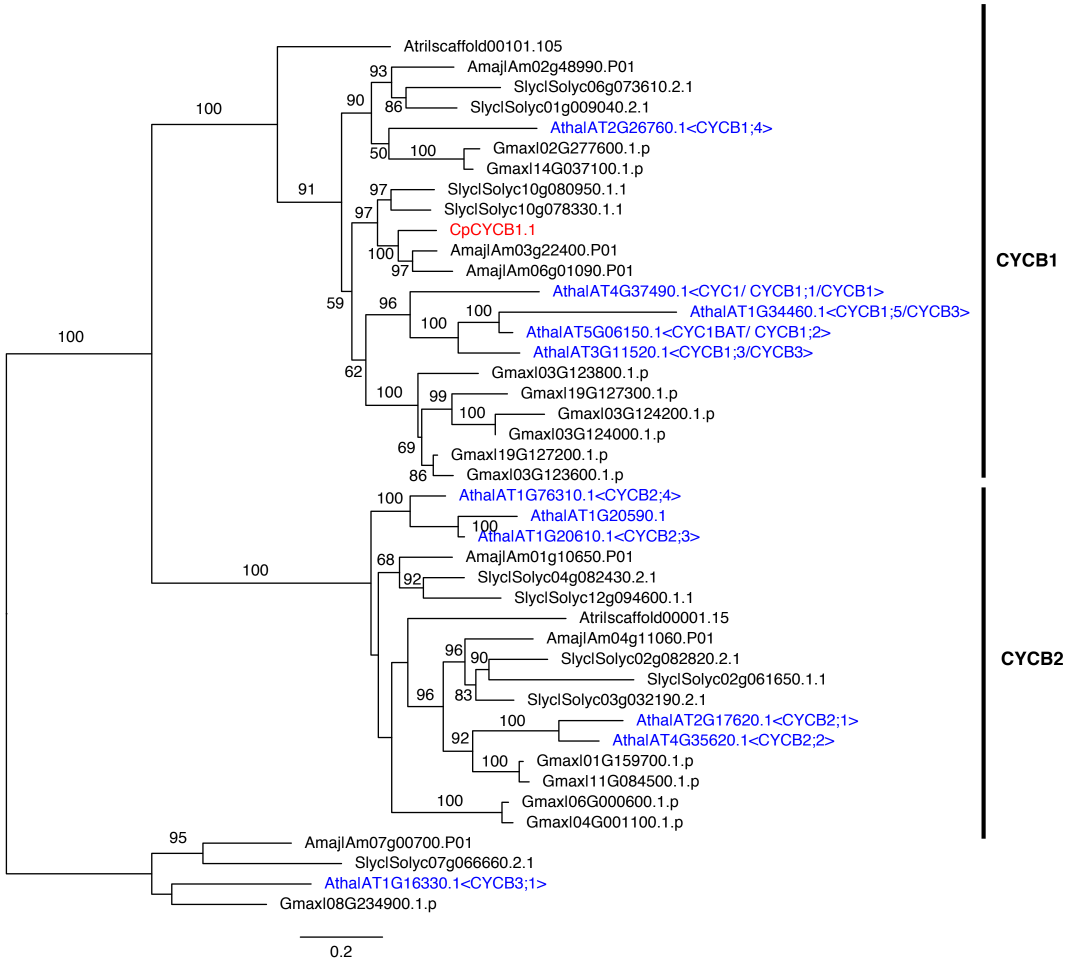

Supplementary Figure 5. **Phylogenetic tree of CYCLIN B proteins.** Numbers at nodes indicate confidence values (%) computed using bootstrapping (1,000 iterations). Values <50% are not shown. Protein sequences were collected from genome databases of five angiosperm species: *Amborella trichopoda* (Atri), snapdragon (Amaj), *Arabidopsis* (Atha), soybeans (Gmax), and tomato (Slyc). Proteins of *Arabidopsis* are shown in blue with their common names and the *C. palustris* protein CpCYCB1.1 is shown in red.

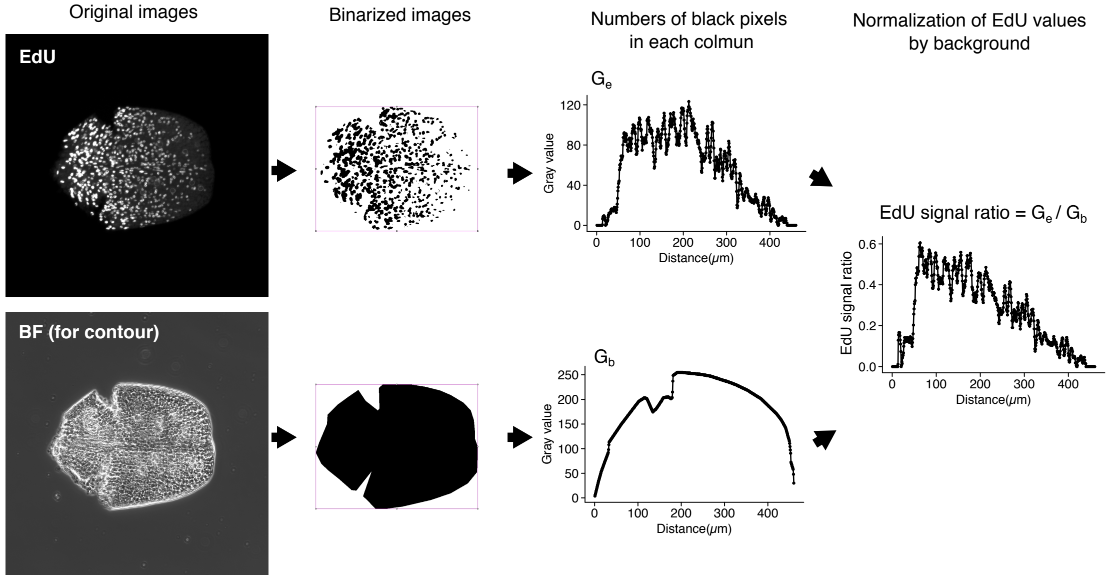

Supplementary Figure 6. Schematic of the quantification of EdU signal intensity. EdU signal intensities along the proximal-distal (left to right in the figure) axis were calculated from a binarized image (top row). Then the raw values were normalized by background values calculated from the leaf silhouette using the same positional information.
